# Research on Surface Integrity and Fatigue Properties in the Turning of TC17 Titanium Alloy Based on the Response Surface Method

**DOI:** 10.3390/ma16227180

**Published:** 2023-11-15

**Authors:** Xunqing Lai, Yuannan Wang, Dan Wang, Guolong Zhao, Yinfei Yang

**Affiliations:** 1 College of Mechanical and Electrical Engineering, Nanjing University of Aeronautics & Astronautics, Nanjing 210016, China; laiexia@nuaa.edu.cn (X.L.); wonderw@nuaa.edu.cn (D.W.); zhaogl@nuaa.edu.cn (G.Z.); 2 Nanjing Mindray Biomedical Electronics Co., Ltd., Nanjing 210019, China; wangyuannan@mindray.com

**Keywords:** TC17 titanium alloy, bending fatigue, turning, response surface, surface integrity, surface roughness

## Abstract

Titanium alloy parts are more and more widely used in the field of aerospace. In order to improve the service life of titanium alloy parts, the response surface method was used to study surface residual stress and roughness under different turning parameters. In addition, a mathematical model was established through multiple linear regression to determine the relationship between surface integrity parameters and fatigue life. The test results indicate that the turning parameters have an effect on surface residual stress in the order of feed rate > depth of cut > cutting speed and on surface roughness in the order of feed rate > cutting speed > depth of cut. The analysis results of surface integrity show that the residual compressive stress on the surface has the greatest impact on fatigue life, followed by surface roughness. The fatigue life increases with the increase in residual compressive stress and decreases linearly with the increase in surface roughness. The feed rate has a significant impact on residual stress and surface roughness. Therefore, under the experimental conditions of this paper, the appropriate feed rate can be selected to ensure that the ${\;R}_{a}$ < 2 μm and a large residual compressive stress is obtained.

## 1. Introduction

Aero-engine reliability mainly depends on the material properties and the surface integrity quality caused by machining technology. The surface damage caused by machining is one of the crucial factors leading to the low reliability and short service time of aero-engines [1]. The materials applied to aircraft engines must first consider service conditions, structural strength, and fatigue performance requirements. Among them, high-temperature titanium alloys, such as TC17, are widely used for their advantages of low density, good thermal strength, and corrosion resistance [2]. However, TC17 is a typical difficult-to-cut material, and its processing parameters are closely related to surface integrity [3,4,5]. Therefore, optimizing and analyzing the turning parameters of TC17 titanium alloy to improve the fatigue life of titanium alloy components is of great engineering and practical significance.

The surface integrity of titanium alloy parts is defined by geometric, mechanical, and metallurgical parameters, which can be expressed by surface roughness and surface residual stress [6]. Many studies have shown the relationship between surface roughness and the fatigue life of titanium alloys. Xiaotong Zhu et al. [7] studied the effect of the surface ultrasonic rolling process on the surface roughness and fatigue properties of TC4 titanium alloy. The test parameters are a vibration frequency of 29 kHz, a current of 1 A, an amplitude of 0.007–0.008 mm, and a rolling ball diameter of 14 mm. The rational speed is 70 r/min, the feed rate is 0.1 mm/rev, and the static pressure is 90 kg. After the rolling process, the maximum value can be reduced to 0.052 μm, and the fatigue strength can be increased by 52% to 425 MPa. Ari et al. [8] studied the relationship between the chip morphology and machining surface integrity of Ti6Al4V alloy in high-speed milling. The results showed that chip morphology can represent the integrity of the machined surface. When the chips are continuously curled, the machining surface is smooth, and the integrity is good. S. Ramesh et al. [9] used the response surface method to optimize the surface roughness of titanium alloy machining. The results showed that the surface roughness increases with an increase in the feed rate but decreases with an increase in the cutting speed and depth of cut. Durul Ulutan et al. [10] summarized the research progress of the machining surface integrity of titanium alloys and believed that surface residual tensile stress is the cause of the surface fatigue failure of materials. Therefore, it needs to be eliminated and prevented. Hongjun Xia et al. [11] studied the influence of micro-milling parameters on the surface roughness of parts, and the research results showed that the feed per tooth had a significant influence. Hirumalai et al. [12] established a prediction model for titanium alloy’s surface roughness and cutting temperature after turning. The experimental results showed that the $f_{z}\;$ had the most significant impact, while the $a_{p}\;$ had a minor impact. Tao Gao et al. [13] studied the influence of the surface integrity and fatigue properties of TC11 titanium alloy, and the results showed that the marks left by turning play a significant role in fatigue fracture, so reducing the surface roughness is the leading research goal. Guolong Zhao et al. [14] studied the relationship between the cutting force, tool wear, and surface damage of composites. The results showed that the tip radius greatly influences the surface roughness and surface damage. Andrzej Matras et al. [15] studied the surface roughness optimization of Ti-6Al-4V titanium alloy, and the results showed that the $v_{c}$ had little effect on the $R_{a}$. Mia, Mozammel, and Grzegorz Krolczyk et al. [16] carried out multi-objective optimization for the turning of Ti-6Al-4V titanium alloys. It was found that a higher cutting speed and lower feed rate have an obvious effect on reducing surface roughness. Kubilay Aslantas et al. [17] used the response surface method to study the influence of machining parameters on the surface roughness of Ti-6Al-4V alloy micro-turning. The overall results show that the feed rate is an important factor affecting the surface roughness. C. -F. Yao et al. [18] studied the relationship between the surface integrity and fatigue life of Ti-10V-2Fe-3Al titanium alloy milling at high speed. The results showed that the fatigue life increases under the given test conditions.

When the surface residual compressive stress increases, the fatigue life of titanium alloy parts is significantly improved [19]. Therefore, the state of residual stress on the surface of titanium alloy is an important factor affecting fatigue life. Y. K. Gao [20] studied the effects of shot peening on the surface roughness and surface residual stress of Ti60 titanium alloy and on the fatigue properties. The results showed that residual compressive stress is conducive to improving fatigue life at room temperature. Neelesh Kumar Sahu et al. [21] used the finite element method-assisted turning test to predict the residual stress of the Ti-6Al-4V alloy. The prediction and test results showed that residual compressive stress is the largest when the cutting speed is 171.4 m/min. Hongmin Xin et al. [22] analyzed the residual stress and affected titanium alloy layer after milling. The results showed that there was residual compressive stress on the milling surface, and the value of this stress decreased with the increase in the spindle speed. Cellier et al. [23] studied the effect of the tool angle on the surface integrity of the Ti6Al4V alloy during milling. It was found that when the axial forward angle was negative, residual compressive stress could be introduced to the machined surface regardless of whether the radial forward angle was negative. Dong Yang et al. [24] studied the relationship between the machining residual stress and cutting parameters of the Ti-6Al-4V alloy. The results showed that the cutting speed and feed rate significantly influenced the cutting residual stress, and the surface residual compressive stress was conducive to extending the fatigue life of parts. Thomas Childerhouse et al. [25] studied the effect of micro-mechanical mechanisms on the fatigue cracks of Ti-6Al-4V carbide cutting tools. The results show that the residual compressive stress caused by machining can restrain the surface defects and enhance the fatigue life of these parts.

Currently, research on the effect of surface roughness and surface residual stress on the turning of process parameters uses finite element analysis algorithms and needs more experimental data as support. For the experimental data obtained, only the effect of a single factor on surface roughness and surface residual stress was considered, while the residual stress and surface roughness resulted from multiple factors. Therefore, based on the study of fatigue properties of titanium alloy, this paper constructs a multi-factor response model through the design of the response surface and reveals the mathematical relationship between the turning process parameters, surface roughness, and surface residual stress.

## 2. Materials and Methods

The test material is TC17 titanium alloy, and the nominal composition is Ti-5Al-2Sn-2Zr-4Mo-4Cr, in which elements Al, Sn, and Zr strengthen the α phase to improve creep resistance, and elements Mo and Cr strengthen the β phase to improve hardenability. The main components are shown in Table 1. The main mechanical properties are shown in Table 2. The base material was 300 × 158 mm in size. The blanks were freely forged in the same batch, and the heat treatment step was heated to 845 °C, held for 4 h, and then water-cooled to room temperature.

**Figure 1:**
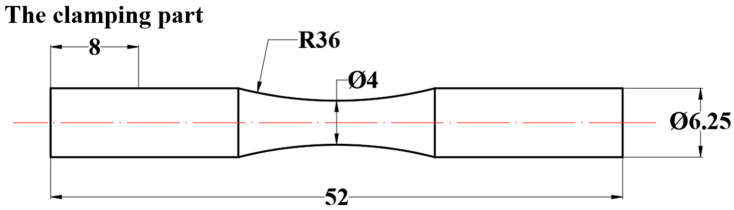
Dimensions of fatigue specimens.

**Figure 2:**
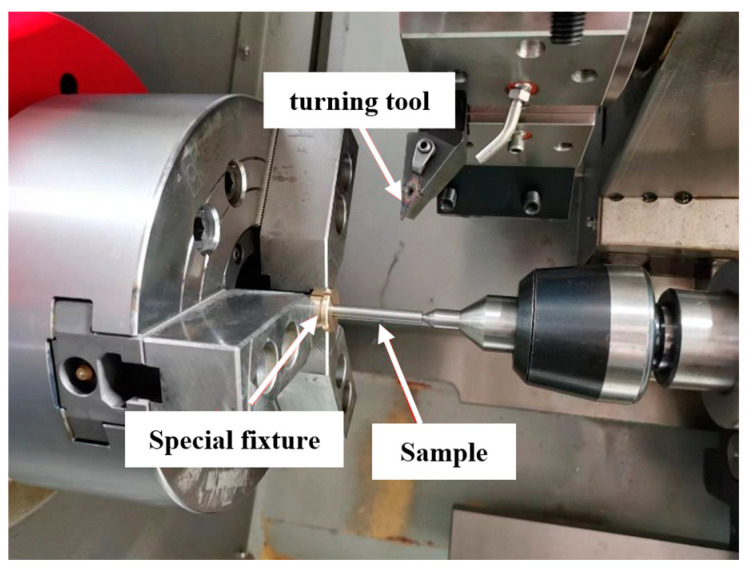
Turning test site diagram.

The size of the test specimen is shown in Figure 1; the length of the gripping part was 8 mm. The turning test site is shown in Figure 2, which was obtained via EDM wire-cutting from the workblank. A DK7732ZT medium-speed electric spark line cutting machine tool was adopted, the dielectric liquid was deionized water, the diameter of molybdenum wire was 0.18 mm, the pulse width was 20 μs, the discharge gap was 6 μm, the number of power amplifier tubes was 6. The outer diameter of the wire-electrode cutting blank was set to be 7 mm.

Then, it reached 6.25 via by external circular fine grinding using a Profimat MT-408 grinding machine produced by BLOHM in Hamburg, Germany. The GC grinding wheel was used. The grinding wheel’s grinding speed was 30 m/s, the grinding depth was 20 μm, and the feed rate was 12 m/min. We used cutting fluid to cool the titanium alloy.

The finishing of the middle area of the specimen was carried out on the CK-3650 horizontal CNC lathe using the tool VNMG160402 and the carbide CVD-coated tool. The corner radius of the cutting tool was 0.2 mm, and the included angle was 35 deg. In order to avoid the impact of tool wear, a new tip was replaced after each specimen was processed. The titanium alloy cutting fluid, which is mainly composed of oil and emulsifier, was sprayed in the cutting area for cooling.

The single-factor test was designed and carried out to optimize the process parameters. In the test, the cutting speed was set to 25 m/min~45 m/min, the feed rate was set to 0.15 mm/rev~0.35 mm/rev, and the depth of cut was set to 0.1 mm~0.5 mm. The design of single-factor test parameters for turning is shown in Table 3.

**Figure 3:**
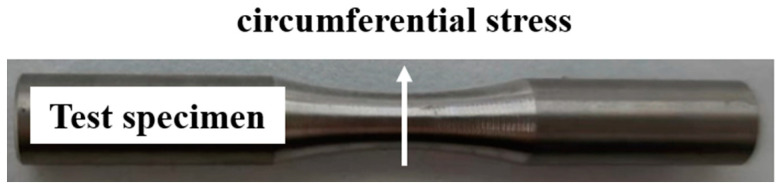
Residual stress measurement direction.

The residual stress on the surface of the test specimen after turning was measured using the μ-X360n X-ray residual stress analyzer made by PULSTEC, Hamamatsu City, Japan. This device uses a full two-dimensional detector and obtains the complete Debye–Scherrer ring based on the method of single-angle incidence. It accurately measures residual stress up to a depth of 10 μm. The residual stress on the surface of the specimen can be measured using the cosα method, and the axial and circumferential stress values of the specimen can be obtained, respectively. Circumferential stress was used for the analysis in this paper. The measuring position was the turning area of the middle arc of the specimen. In order to reduce the error, all the results are the average value after three measurements. The measurement position and stress direction are shown in Figure 3.

**Figure 4:**
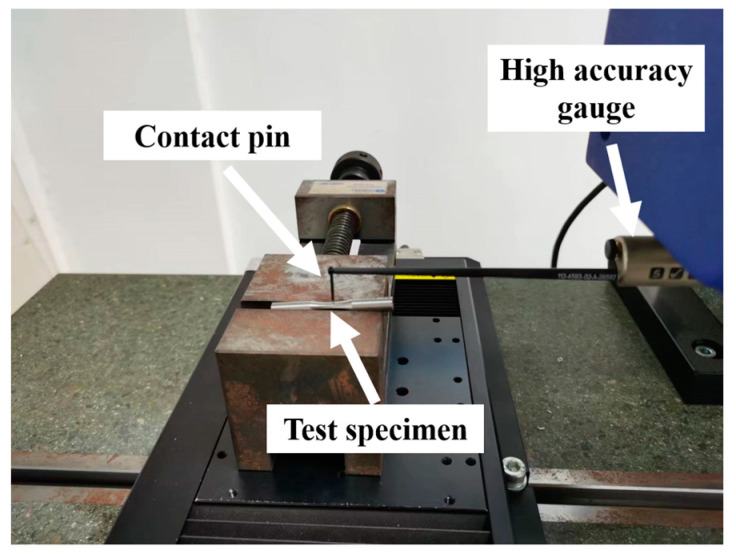
Roughness measurement diagram.

According to the characteristics of the turning itself, the surface roughness in the feed direction was selected as the research object for analysis. The surface roughness measurement site is shown in the Figure 4. Roughness was measured using the Form Talysurf i-serious Roughness Profiler from TAYLOR HOBSON, London, UK. The roughness measurement standard is referred to as GB/T 10610-2009 [27]. When 2 < $R_{a}$ ≤ 10, the sampling length was 2.5 mm, and the evaluation length was 12.5 mm. The measuring position was the turning area of the middle arc of the specimen and the average value after three measurements.

**Figure 5:**
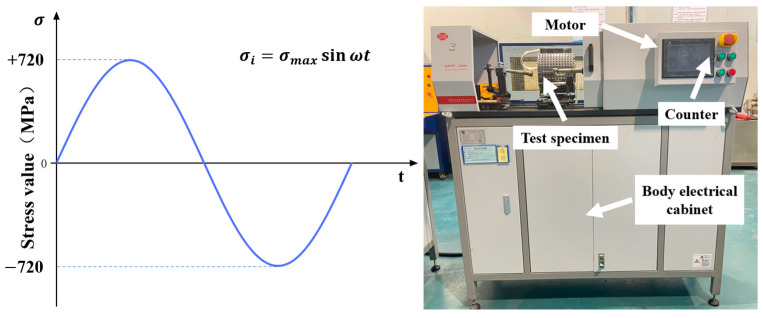
Stress cycle characteristics and bending fatigue test equipment.

The fatigue test was carried out on the QBWP-1000 cantilever bending fatigue test machine. The fatigue test conditions are shown in Table 4, and the stress cycle characteristics of the bending fatigue test equipment are shown in Figure 5. The median fatigue life data obtained were taken as the final fatigue test data.

## 3. Results

### 3.1. Single Factor Test Results

**Figure 6:**
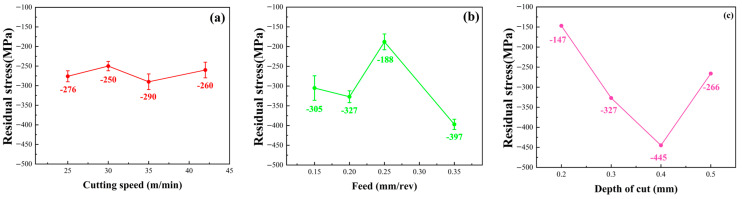
Residual stress of machined TC17 under various turning parameters. (**a**) Residual stress at different cutting speeds, (**b**) Residual stress at different feed rates, (**c**) Residual stress at different depths of cut.

As shown in Figure 6, the influence law of turning process parameters on surface residual stress mainly presents residual compressive stress, which is conducive to improving the fatigue life of the workpiece. The mean surface residual compressive stress after turning is −280 MPa, and the surface residual stress is more sensitive to the changes in f and $a_{p}$ than the $v_{c}$.

**Figure 7:**
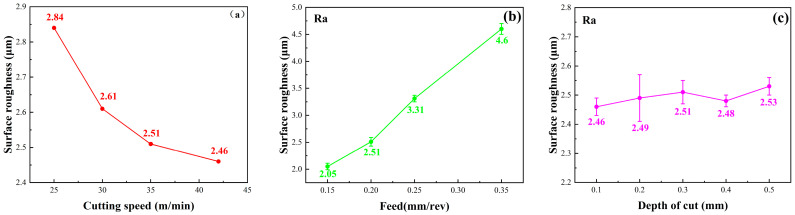
Surface roughness of machined TC17 under various turning parameters. (**a**) Surface roughness at different cutting speeds, (**b**) Surface roughness at different feed rates, (**c**) Surface roughness at different depths of cut.

The measurement results of the middle turning part of specimens are shown in Figure 7. The surface roughness ($R_{a}$) of the rest of the specimen is 0.7~0.8 μm. It can be seen that increasing the $v_{c}$ and decreasing the f is conducive to a reduction in the surface roughness. The surface roughness increases with the increase in $a_{p}$, but the overall variation is small.

Based on the results of the single-factor test, the cutting parameters that could ensure the surface roughness would be small were selected first, and the parameter range that could ensure the surface residual compressive stress would be large was considered. Finally, a new cutting parameter domain was selected. The effect of the turning process parameters on the surface roughness value and surface residual stress changes was further studied. As shown in Table 5, three factors and three levels were selected in the field of optimal process parameters, and the relationship between each turning process parameter within the range and each element of surface integrity was intuitively studied through the design response surface test.

### 3.2. Analysis and Modeling Based on Response Surface Method

#### 3.2.1. Response Surface Method Test Results

Based on the above test parameters, the Box–Benhnken design response surface method was used for the turning test. After the test, the surface roughness and residual stress of the test specimen were measured by the surface profile and residual stress detector above, respectively. Table 6 provides the accurate test parameters and corresponding measurement data.

#### 3.2.2. Analysis of Variance (ANOVA) of Experimental Results

The ANOVA results of surface residual stress after turning are shown in Table 7. Through the analysis of variance, we can see the degree of influence of each factor on the response value. Adj SS is a measure of the deviation of different parts of the model. Adj MS measures the degree to which a term or model explains variability. The *p*-value is a value that measures the significant difference, requiring *p* < 0.05 for the model item and *p* > 0.05 for the lack of fit. The F-value is a test statistic that is used to determine whether any item in the model is associated with the response [28]. From the F-value of the primary term, it can be seen that the order of significance of the turning factors affecting the surface residual stress is f > $a_{p}$ > $v_{c}$.

The regression coefficient evaluation of surface roughness after turning and the corresponding significance test results are shown in Table 8. In addition, from the F value of the primary term coefficient, it can be seen that the order of significance of turning factors affecting the surface roughness is f > $v_{c}$ > $a_{p}$. The feed rate has a great influence on the surface roughness. The f has a great effect on the surface roughness, while the $a_{p}$ has no significant effect.

#### 3.2.3. The Establishment of the Response Mathematical Model

The multiple quadratic regression equation is obtained by fitting the experimental data [29,30], and then the optimal process parameters are sought by analyzing the multiple quadratic regression equation. The general form of the quadratic regression equation obtained using the response surface method is as follows:

(1) $$y=\gamma_{0}+\sum_{i=1}^{n}\gamma_{i}x_{i}+\sum_{i=1}^{n}\gamma_{i}x_{i}^{2}+\sum_{i=1}^{n}\sum_{j=1}^{n}\gamma_{ij}x_{i}x_{j}$$

In Equation (1), y is the response value with respect to x,$\;\;\gamma_{0},\gamma_{i},\;\;\gamma_{ij}$ are constants, $x_{i}$ and $x_{j}$ are the i and j independent variables, and n represents the number of parameters.

With the help of statistical analysis software Design-Expert v10.0, experimental data in Table 6 were fitted via multiple linear regression to obtain the mathematical response model of the residual stress (σ) and surface roughness (Ra) of turning as follows:

(2) $$\begin{array}{cc} & \sigma =-589.63125+6.4075v_{c}+2778.25f\\ & -350.25a_{p}-6.75v_{c}f+0.75v_{c}a_{p}\\ & +675a_{p}f-0.05025v_{c}^{2}-5502.5f^{2}+147.5a_{p}^{2} \end{array}$$

(3) $$\begin{array}{cc} & R_{a}=0.13347-0.027119v_{c}+26.66234f\\ & +6.72520a_{p}+0.00575v_{c}a_{p}-1.25a_{p}f\\ & -15.84737f^{2}-19.00987a_{p}^{2} \end{array}$$

**Figure 8:**
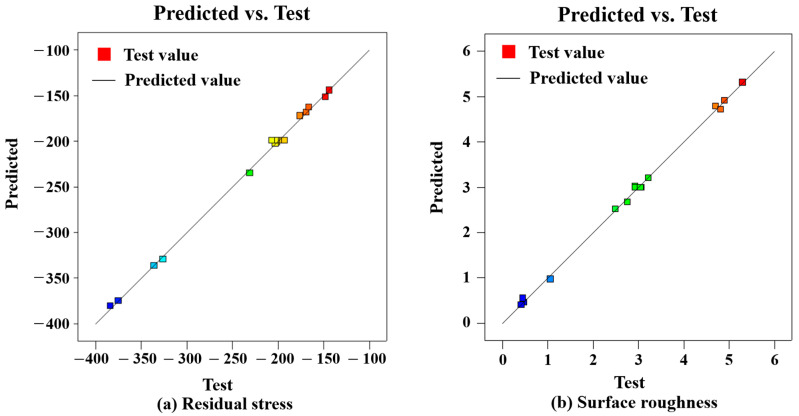
Comparison of predicted models for surface roughness and residual stress. Different colors in the figure indicate the fatigue life of the specimen corresponding to different residual stress and roughness values.

The comparison between the predicted and measured values of the surface residual stress (a) and surface roughness (b) in Figure 8 was obtained via the fitting of Formulas (2) and (3). The black line represents the predicted value of the model, and the square represents the test value. It can be seen from the figure that the predicted values of the surface residual stress and surface roughness are relatively consistent with the measured values. It shows that the model fits well with the actual results of the text.

### 3.3. TC17 Turning Parameter Interaction Response Surface Graph Analysis

**Figure 9:**
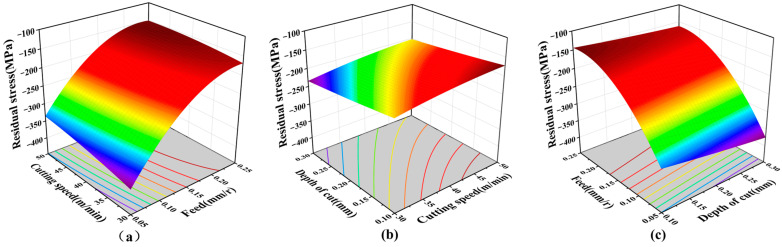
The influence of the machining factor on residual stress. (**a**) Interaction of $v_{c}\;and\;f$ on residual stress, (**b**) Interaction of ${\;v}_{c}\;and\;a_{p}$ on residual stress, and (**c**) Interaction of$\;f\;and\;a_{p}$ on residual stress.

Combined with the data results in Table 6, the response surface diagram between the response value and the input value was drawn, the influence law of the interaction of the two factors on the residual stress was analyzed, and the process parameters were optimized. Figure 9a shows the residual stress response surface diagram under the interaction action of $v_{c}$ and f at a fixed $a_{p}$ of 0.2 mm. As can be seen from the figure, with the increase in f, the surface residual compressive stress showed a trend of decreasing gradually. This indicates that an appropriate reduction in f is conducive to increasing the surface residual compressive stress, and an appropriate surface residual compressive stress is conducive to improving the fatigue life of the workpiece [31]. However, the interaction effect of the linear velocity and feed rate on surface residual stress is not significant. As shown in Figure 9b, with the increase in $a_{p}$, the value of residual compressive stress showed an increasing trend, but this change was relatively gentle. When $a_{p}$ reached 0.3 mm, the residual compressive stress reached its peak. It can be seen from Figure 9c that the surface residual stress was more sensitive to the change in f when the fixed $v_{c}$ was 40 m/min. When f increased from 0.2 mm/rev to 0.25 mm/rev, the residual compressive stress decreased significantly. In general, a smaller f and larger $a_{p}$ are conducive to increasing the residual compressive stress.

**Figure 10:**
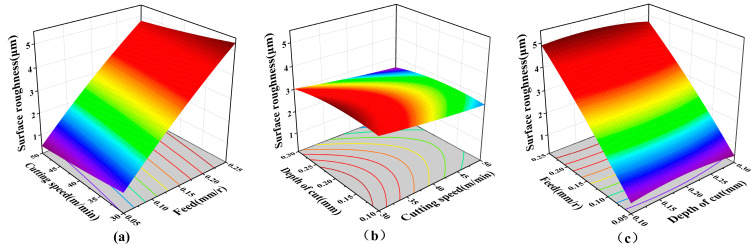
The influence of the machining factor on surface roughness. (**a**) Interaction of $v_{c}\;and\;f$ on surface roughness, (**b**) Interaction of${\;v}_{c}\;and\;a_{p}$ on surface roughness, and (**c**) Interaction of$\;f\;and\;a_{p}$ on surface roughness.

As shown in Figure 10a, by analyzing the mathematical model of surface roughness, when the fixed $a_{p}$ was 0.2 mm, the surface roughness was very sensitive to the change in f. With the increase in f, the surface roughness also increased, almost showing a linear change. In the range of experimental parameters, with the increase in $v_{c}$, surface roughness had a certain optimization effect. As shown in Figure 10b, when the fixed f was 0.15 mm/rev, the surface roughness was not sensitive to the interaction between $a_{p}$ and $v_{c}$, and the surface roughness remained almost unchanged. As shown in Figure 10c, $a_{p}$ had little influence on the surface roughness. In summary, for the range of test parameters, reducing f can effectively reduce the surface roughness, and increasing the $v_{c}$ can appropriately reduce the surface roughness.

### 3.4. TC17 Fatigue Life Model

**Figure 11:**
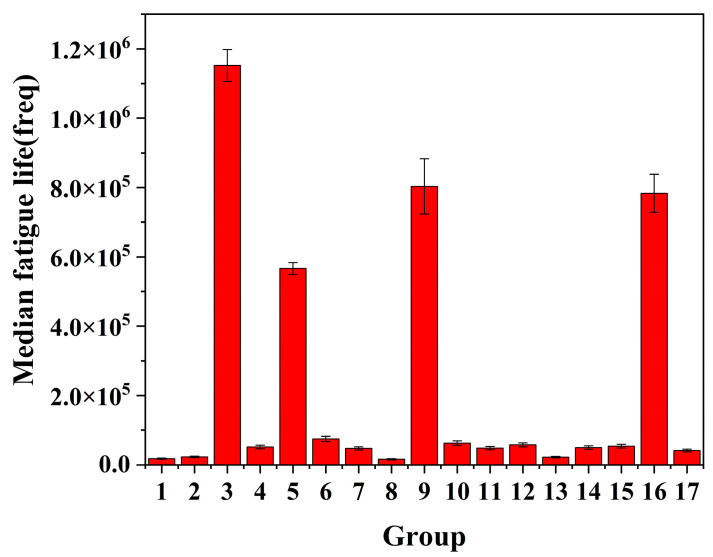
Fatigue life of TC17 under different fatigue parameters.

The samples processed with different turning parameters were tested on the cantilever bending fatigue test machine with the parameters in Table 6. The fatigue life is shown in Figure 11, where 1 to 17 in the Figure are seventeen groups of parameters, with each group of parameters processing three fatigue test specimens as the mean fatigue life. It can be seen from the figure that the fatigue life of the TC17 titanium alloy at room temperature is generally more than 10,000 cycles, and the data are relatively discrete. According to the median fatigue life of Figure 9b, it can be seen that the fatigue life under the third group of turning parameters is about 65 times that under the first group of turning parameters.

**Figure 12:**
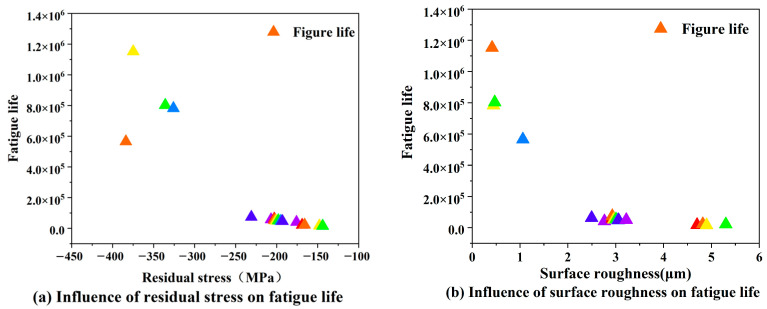
Influence of surface integrity on fatigue life. Different colors in the figure indicate the fatigue life of the specimen corresponding to different residual stress and roughness values.

The relationship between surface integrity elements and fatigue life can be obtained by combining Table 6 and Figure 11. As shown in Figure 12, the fatigue life decreases significantly with the increase in surface roughness. When $R_{a}$ > 2 μm, the fatigue life of TC17 is less than $10^{5}$ times. This is because, after the turning process, the surface defects increase, which leads to deeper surface micro-grooves, which correspond to a more serious local stress concentration, promoting the initiation of surface micro-cracks, and the surface micro-cracks begin to expand until the test rod breaks during the fatigue test. The fatigue life clearly increases with the increase in surface residual compressive stress. The fatigue life is above five times, especially when the absolute value of the surface residual compressive stress is greater than 250 MPa, and the fatigue life is above 5$\;{\times \;10}^{5}$ times. This is because when the appropriate surface residual compressive stress is superimposed on the fatigue load, the average stress in the fatigue test changes, thus affecting the fatigue life.

In the process of turning surface formation, the coupling effect of surface integrity factors on the fatigue life of TC17 titanium alloy test parts should be considered comprehensively. Many fatigue prediction methods exist, such as artificial neural networks and finite element simulation [32,33]. In this paper, based on the above experimental data, linear regression analysis was used to establish the mathematical model between fatigue life $N_{f}$, surface residual stress, and surface roughness.

Data are assembled in Table 6 and Figure 9. The mathematical model between the fatigue life and surface integrity parameters of specimens was obtained using a linear regression method. The empirical formula is as follows:

(4) $$N_{f}=A_{0}\sigma_{r}^{a}{Ra}^{b}$$

In the formula, $A_{0}\;$is a constant. The existing data are fitted by origin, and the correlation coefficient of linear regression is 0.90836 according to the fitting result of the least square method. The fitting of the empirical formula was obtained by plugging the fitting value into Formula (4):

(5) $$N_{f}=0.4999|\sigma_{r}{|}^{2.3106}{Ra}^{-0.8199}$$

According to the empirical formula, the surface residual stress has the greatest effect on the fatigue life, while the surface roughness has a relatively small effect. The surface residual stress can be increased by appropriately reducing the f. The roughness increases with the increase in f. Therefore, according to the test results, within the parameter range of the test, f should be less than 0.1 mm/rev, $v_{c}$ should choose a smaller value, such as 30 m/min, and $a_{p}$ can be selected at 0.3 mm because $a_{p}$ has the smallest impact on the residual stress and surface roughness.

### 3.5. Analysis of Fatigue Fracture

**Figure 13:**
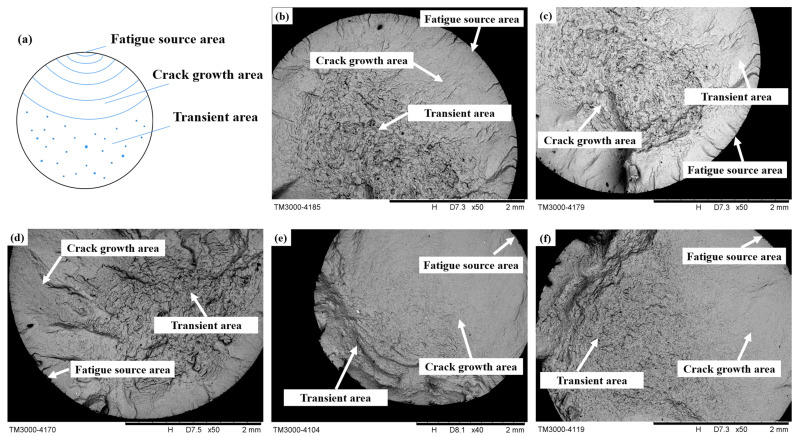
Macroscopic fatigue fracture diagram. (**a**) Fatigue crack growth schematic diagram, (**b**) Microscopic morphology of the fatigue fracture of the first group of specimens, (**c**) Microscopic morphology of fatigue fracture of the second group of specimens, (**d**) Microscopic morphology of fatigue fracture of the third group of specimens, (**e**) Microscopic morphology of fatigue fracture of the fourth group of specimens, (**f**) Microscopic morphology of fatigue fracture of the fifth group of specimens.

The fatigue crack propagation of the specimen can be divided into three regions, as shown in Figure 13a: the fatigue source area, crack growth area, and transient area. This figure shows the macroscopic morphology of the fatigue fracture surface of the TC17 specimen in this experiment, and it can be clearly seen that the fatigue fracture surface has similar characteristics. Three regions of crack propagation can be observed from the fracture surface. It can be seen that the cracks in the specimen originate from surface defects after turning. This is due to the high surface stress level of the specimen in this test. In high-roughness specimens, fatigue cracks are prone to initiate machined surface defects. Then, they expand radially toward the surroundings. When the remaining part cannot withstand the applied load, the specimen instantly fractures.

**Figure 14:**
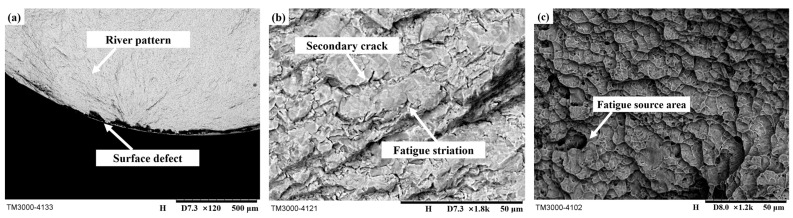
Microscopic fatigue fracture diagram. (**a**) Fatigue source area, (**b**) Crack growth area, (**c**) Transient area.

Figure 14 shows the microstructure of the fatigue fracture surface. Figure 14a shows the microscopic morphology of the fatigue source area, which shows apparent brittle fracture characteristics. River patterns can be observed near the fatigue source area. Figure 14b shows the microscopic morphology of the crack growth area, with obvious secondary cracks and fatigue bands. This is because the TC17 titanium alloy used in the experiment is a basket structure, and the α phase and β phase stress concentrations occur at the junction of phases. Figure 14c shows the microscopic morphology of the transient area. Due to the presence of numerous dimples observed in this area, the fracture in this area exhibits the microscopic characteristics of ductile fracture.

## 4. Conclusions

In this paper, the fatigue test of the TC17 titanium alloy smooth sample was carried out, the single-factor test was designed, and the three-factor response surface test was further carried out. The turning surface integrity and fatigue life parameters were obtained. The multi-factor to multi-factor response mathematical model between the turning parameters and surface integrity was constructed using the response surface method. The effects of surface roughness and surface residual stress on fatigue life were established via linear regression. The empirical formulas of surface integrity and fatigue life were obtained by fitting the least square method, and the main conclusions are summarized as follows:

1. A response model of turning parameters, surface residual stress, and surface roughness was constructed using the design–expert v10.0 software. According to the response surface graph and variance analysis table, it was found that the sequence of significance of the turning parameters affecting surface residual stress was as follows: f > $a_{p}$ > $v_{c}$; the order of significance of turning parameters that affect surface roughness is f > $v_{c}$ > $a_{p}$. Under the processing conditions of this paper, the range of residual stress was −148 MPa to −384 MPa, and the range of surface roughness was 0.42 μm to 5.3 μm. When $v_{c}$ is 40 m/min, f is 0.05 mm/rev and $a_{p}\;$is 0.3 mm, and the surface roughness is the minimum. When $v_{c}$ is 30 m/min, f is 0.05 mm/rev and $a_{p}\;$is 0.2 mm, and residual compressive stress is the maximum.
2. Based on the fatigue life test results of specimens, the residual stress and surface roughness of the machined surface has a significant influence on the fatigue life of the sample, and the fatigue life decreases linearly with the increase in surface roughness. When the machining of residual compressive stress is introduced into the machined surface, the fatigue life can be significantly improved. The most extended fatigue life is 1,198,070 times, while the shortest fatigue life is only 14,470 times, and the span of fatigue life is large.
3. The influence of the law between various elements of surface integrity and fatigue life was studied, and the mathematical equation between the two was fitted using the least square method. It was concluded that the surface residual compressive stress had the greatest influence on fatigue life, followed by surface roughness. Therefore, the selection of turning parameters should be combined with the changing trend of residual compressive stress and surface roughness, and f should be increased appropriately while ensuring surface roughness. When 30 m/min ≤${\;v}_{c}\;$≤ 50 m/min, 0.1 mm ≤${\;a}_{p}\;$≤ 0.3 mm, f should not exceed 0.15 mm/rev.

## Figures and Tables

**Table 1:** The material composition of TC17 [26].

Ti	Al	Sn	Zr	Mo	Cr	N	Fe	C	O	Others
Bal.	4.9	2	1.9	4.2	4.2	0.02	0.1	0.02	0.1	Each < 0.10
										Total < 0.30

**Table 2:** The mechanical property of TC17 [26].

$\sigma_{b}$ (MPa)	$\sigma_{0.2}$ (MPa)	Density $(Kg/m^{3}$)	Shrinkage (%)	Elongation (%)	Conductivity W(m·°C)	Expansion Coeff $(10^{-6}/$°C)
1180	1110	4770	17.5	10	6.21	11.8

**Table 3:** Single-factor turning test parameters.

	$v_{c}$ (m/min)	f (mm/rev)	$a_{p}$ (mm)
Group I	25, 30, 35, 42	0.15	0.1
Group II	35	0.15, 0.2, 0.25, 0.35	0.1
Group III	35	0.2	0.1, 0.2, 0.3, 0.4, 0.5

**Table 4:** Rotary bending fatigue test parameters.

Experimental Parameter	$\sigma_{max}$ (Mpa)	*r*	*f* (Hz)	$T_{room}$ (°C)	Waveform	The Form of Failure
Set value	720	−1	83.3	20	Sine wave	Working section fracture

**Table 5:** Process parameter level.

Code	$v_{c}$ (m/min)	f (mm/rev)	$a_{p}$ (mm)
A	30	0.05	0.1
B	40	0.15	0.2
C	50	0.25	0.3

**Table 6:** Response surface experimental results.

No.	$v_{c}$ (m/min)	f (mm/rev)	$a_{p}$ (mm)	Ra (μm)	σ (MPa)
1	50	0.25	0.2	4.7	−148
2	40	0.25	0.3	4.82	−166
3	40	0.05	0.3	0.42	−375
4	40	0.15	0.2	2.96	−198
5	30	0.05	0.2	1.06	−384
6	30	0.15	0.3	2.93	−231
7	40	0.15	0.2	3.05	−193
8	40	0.25	0.1	4.9	−144
9	50	0.05	0.2	0.47	−336
10	50	0.15	0.3	2.5	−203
11	40	0.15	0.2	3.06	−195
12	40	0.15	0.2	2.93	−207
13	30	0.25	0.2	5.3	−169
14	30	0.15	0.1	3.22	−201
15	40	0.15	0.2	3.01	−203
16	40	0.05	0.1	0.45	−326
17	50	0.15	0.1	2.76	−176

**Table 7:** Analysis of variance for residual stress.

Source	DF	Adj SS	Adj MS	F-Value	*p*-Value	Significant
Model	9	96,107.76	10,678.64	360.591	<0.0001	YES
A	1	1860.50	1860.50	62.824	<0.0001	
B	1	78,804.50	78,804.50	2661.030	<0.0001	
C	1	2048.00	2048.00	69.156	<0.0001	
AB	1	182.25	182.25	6.154	0.0229	
BC	1	182.25	182.25	6.154	0.0229	
A^2^	1	106.32	106.32	3.590	0.01	
B^2^	1	12,748.42	12,748.42	430.482	<0.0001	
Residual	7	207.30	29.61			
Lack of Fit	3	74.50	24.83	0.748	0.7565	
Pure Error	4	132.80	33.20			
Cor Total	16	96,315.06				NO
R-squared		0.9997				

**Table 8:** Analysis of variance for surface roughness.

Source	DF	Adj SS	Adj MS	F-Value	*p*-Value	Significant
Model	9	38.40	4.27	438.624	<0.0001	YES
A	1	0.54	0.54	55.456	<0.0001	
B	1	37.53	37.53	3857.336	<0.0001	
C	1	0.06	0.06	5.751	0.0305	
AC	1	0.00	0.00	0.014	0.0091	
BC	1	0.00	0.00	0.064	0.0087	
B^2^	1	0.11	0.11	11.196	0.0123	
C^2^	1	0.16	0.16	16.032	0.0052	
Residual	7	0.07	0.01			
Lack of Fit	3	0.06	0.02	5.803	0.0612	
Pure Error	4	0.01	0.00			
Cor Total	16	38.47				NO
R-Squared	0.9980					

## Data Availability

Data are contained within the article.
